# Supplementary material for: Reversible neuropathic pain model created by long-term optogenetic nociceptor stimulation using light-responsive pain mice
Source: PLoS One. 2025 May 30;20(5):e0323628. doi: 10.1371/journal.pone.0323628 (PMC12124532; doi:10.1371/journal.pone.0323628)
Supplement: Attachment — Submitted filename: reviewing Kouroki et al.pdf [file pone.0323628.s002.pdf]

Reversible neuropathic pain model created by long-term optogenetic nociceptor stimulation using light-responsive pain mice.

Satoshi Kouroki et al.

The authors are addressing an important issue that is the lack of treatment of neuropathic pain and its subsequent societal impact and how having better animal models could help define novel therapeutic targets and strategies.
They therefore aim at understanding the "pure nociceptive" component of pain without other components usually associated to it in other animal models. To this end, they take an optogenetic approach to stimulate nociceptors expressing different types of voltage-gated sodium channels. Experiments are well executed; however, the manuscript could be largely improved in terms of writing to better describe the aim and conclusions of the experiments. A few key experiments are also lacking to fully support their conclusions.

1. The use of "neuropathic pain" in the description of their model is confusing. Indeed, neuropathy is large family of pain types which can be acute, a chronic state resulting from neuronal lesion with sensory alterations, combined to inflammation or metabolic alterations... In the discussion, they compare the advantage of their acute model of nociception over sciatic nerve injury that leads to chronic neuropathy, a very different clinical picture. A more precise description of the proposed model in respect to clinical relevance therefore needs to be clarified to draw the right conclusions.

In this line, the authors should give examples and references about the other animal models of neuropathic pain they are comparing theirs to (introduction, line 53) to clarify latter the potential added value and limitations of their model. Also, they refer to a previous work of theirs introducing this optogenetic-based model (introduction, line 61-65) and mention that "nociceptive pain was produced", not neuropathic pain….

2. To comprehend the importance of each sodium channel they investigate, it is also important to describe better in the introduction their expression pattern in the nociceptive and sensory fiber types, their difference in terms of electrophysiology (for instance their different sensitivity to TTX), and the state of the art knowledge about their implication in neuropathic pain as their involvement is not utterly understood. Their role also needs to be described in line with the type of pain elicited by this optogenetic-induced nociceptor stimulation.

3. Description of the results should be better written.
Replace "Fig. X shows" throughout the section. For instance, on page 20 you should state "we investigated/examined c-Fos expression" instead of "we observed c-Fos expression" and then "we observed" or "this revealed" instead of "Fig.3 shows".

4. On page 21, there is no introduction about the aim of the experiment. Why studying DS-1971a effect? Why focusing on NaV1.7 and not investigating the others? Why looking at the responses of the NaV1.8 and NaV1.9 mice after DS-1971a treatment if NaV1.7 is "globally expressed"? This also goes back to better describing the expression and role of these three sodium channels in the introduction.

Your description of the results (lines 241-243) is a title of a figure legend but definitively not a result description/explanation. This must be rephrased.

Additional experiments should also be done to fully support your conclusions mentioned in the discussion.
It is important to examine the long-lasting effect of the NaV1.7 inhibitor using the same treatment paradigm. Given your model elicits allodynia for 24 h, does NaV1.7 inhibition prevent hypersensitivity appearance when looking at 24 h post-optogenetic stimulation or does it only block the acute phase you investigated here and simply delays sensory alterations?
In this line, NaV expressions are altered in neuropathic pain, it would be of interest to assess whether this is the case in your model 3 h, 24 h, and 48 h after light simulation during the acute phase when DS-1971a is effective, the subacute phase (to link it with the (lack of) effect of DS-1971a at that time point), and when normal sensory response is restored, respectively.
Additionally, given DS-1971a is efficient in all mouse genotypes, you would gain crucial information i/ by examining the influence of DS-1971a in hyperalgia tests in addition to allodynia and ii/ by specifically discussing the role of A-beta sensory fibres, and C vs A-delta nociceptive fibers as the three NaV channels have different expression patterns.
In this line, it would be important to test the impact of low and high TTX doses to discriminate A fibre (low dose) from A+C fibre (high dose) implications in nociceptive and sensory tests.
Also, does blocking NaV1.8 using VX-548 or LTGO-33 for instance lead to the same result in all three genotypes in different nociceptive and sensory tests?

This is crucial for the description of your model, for the understanding of the role of each sodium channel, thereby for proposing specific therapeutic targets for specific pain types.

Minor comments

1. c-Fos expression is not a pain perception marker as stated several times in the manuscript. It reflects neuronal activity. In this case linked to nociceptor stimulation yet its expression is not specific to pain. This has therefore to be modified.

2. Below-mentioned sentences in introduction (lines 65-67) belong to the discussion or need to be rephrased:
“Previously, Daou et al. generated NaV1.8−ChR2 mice using the NaV1.8−Cre recombinase knock-in mouse line and reported results similar to ours [10]. They also reported the occurrence of transient neuropathic pain following long-term exposure to blue light.”

3. Materials & Methods, line 83. The authors mention that the experimenters are blinded to the mouse genotypes. Are they also blinded to the treatments (DS-1971a vs vehicle) used as this is also crucial?

4. Materials & Methods, lines 149-151. The below-mentioned sentence goes into the results section:

“Mice were confirmed hypersensitive by von Frey test 1 hour after a prolonged (30 min), suprathreshold (7.5 mW for Nav1.7–ChR2, 1.8 mW for NaV1.8–ChR2, and 2.25 mW forNaV1.9–ChR2) light stimulation.”

5. Results. Line 208. “Continuous” should be replaced by “prolonged”.

6. Discussion is lacking an introductory a summary of your findings.

7. Discussion (lines 276-279). This belongs to the result section.

8. Discussion (line 286). Please give references.
