# Supplementary material for: Reversible neuropathic pain model created by long-term optogenetic nociceptor stimulation using light-responsive pain mice
Source: PLoS One. 2025 May 30;20(5):e0323628. doi: 10.1371/journal.pone.0323628 (PMC12124532; doi:10.1371/journal.pone.0323628)
Supplement: Attachment — Submitted filename: Reviewing R1_PLoS One.docx [file pone.0323628.s004.docx]

**While the authors addressed most of the concerns, their model clearly depicts central sensitization that may lead to neuropathic pain but does not convincingly show it recapitulates clinical features of neuropathic pain (and consequently that it is model of neuropathic pain in comparison to the Chung model or a diabetic model for instance), nor that it shows advantages over other existing models. As this is mostly semantic, changes can be easily made to improve the precision in the manuscript. However, a deeper understanding of the role of NaV1.7 and a comparison with that of NaV1.8 in the generation of central sensitization need to be experimentally addressed to increase the interest of their model and provide added value to the existing literature.**

**Please find below the comments to their responses.**

Reviewer: 1

Major comments:

1) The use of “neuropathic pain” in the description of their model is confusing. Indeed, neuropathy is large family of pain types which can be acute, a chronic state resulting from neuronal lesion with sensory alterations, combined to inflammation or metabolic alterations... In the discussion, they compare the advantage of their acute model of nociception over sciatic nerve injury that leads to chronic neuropathy, a very different clinical picture. A more precise description of the proposed model in respect to clinical relevance therefore needs to be clarified to draw the right conclusions.

In this line, the authors should give examples and references about the other animal models of neuropathic pain they are comparing theirs to (introduction, line 53) to clarify latter the potential added value and limitations of their model. Also, they refer to a previous work of theirs introducing this optogenetic-based model (introduction, line 61-65) and mention that “nociceptive pain was produced”, not neuropathic pain….

**Response:** Thank you for your valuable comment. We have already provided examples and references about the other animal models of neuropathic pain, which we are comparing our model to, to clarify latter the potential added value of our model in the discussion section. Kindly see Discussion, Pages 27–29: lines 311–327, “A multitude of animal models of neuropathic pain…specific voltage-gated Na+ channels”.

The authors did not include any example nor references of other models of neuropathic pain in the introduction (as previously asked) to later compare the supposed advantages of their model.

Indeed, in the discussion, they did not get into the specificities of their model vs others in respect to the fact that existing models “may not accurately recapitulate the clinical manifestations of the disease”. I still do not see how their model addresses this question. I understand their model produces central sensitization (which may lead to neuropathic pain) and that the model can be of importance for studying the initial mechanisms of central sensitization, but there is no data supporting that this is a clinically relevant model of neuropathic pain, notably because neuropathic pain is a chronic state (for instance in the Chung model which is the only one they discuss) while sensory alterations are reversible after 24 hours in their model of acute neuropathy.

Our light-responsive pain mice (NaV1.x−ChR2) induce nociceptive pain on exposure to specific wavelengths, similar to the light-responsive pain mice (NaV1.8−ChR2) developed by Daou et al. Moreover, repeated nociceptive pain can lead to neuropathic pain due to central sensitization [16]. Daou et al. and we have also demonstrated that prolonged light exposure can cause neuropathic pain. We have already mentioned these points in the discussion section (Page 26: lines 294-301,“Daou et al. reported that…thereby leading to central sensitization [16]”).

2) To comprehend the importance of each sodium channel they investigate, it is also important to describe better in the introduction their expression pattern in the nociceptive and sensory fiber types, their difference in terms of electrophysiology3) (for instance their different sensitivity to TTX), and the state of the art knowledge about their implication in neuropathic pain as their involvement is not utterly understood. Their role also needs to be described in line with the type of pain elicited by this optogenetic-induced nociceptor stimulation.

**Response:** Thank you for your valuable comment. As you have accurately pointed out, in order for the reader to understand the importance of the Na+ channels we are studying, the expression pattern of each Na+ channel, their electrophysiological differences and the latest findings on their involvement in neuropathic pain should be explained. However, these findings are lengthy and would better be summarized in a review article, and cannot be explained in the introduction; we have already mentioned the role of Na+ channels, citing the review by Wada et al. [3] (Pages 5–6: lines 55–57), and further we have added text on Na channels (Pages 6–7: lines 57–70) with an additional reference [4]. Readers of this paper will probably also read the preceding paper [12]. As the role of the Na channel is also described in that preceding paper, we feel that the description of the Na+ channels in this paper is sufficient.

**Changes in the text:**

“In the voltage-gated Na+ channels, which consist of NaV1.1-1.9 subtypes, each subtype possesses different kinetics and expression patterns, which are reflected in the functional groupings of the peripheral sensory neurons [4]. Large DRG neurons with myelinated Aβ and Aδ fibers express mainly tetrodotoxin-sensitive (TTX-S) NaV1.1, NaV1.6, and NaV1.7. A subset of these neurons also express tetrodotoxin-resistant (TTX-R) NaV1.8, which may correspond to Aβ nociceptors. In contrast, small diameter nociceptive neurons with unmyelinated C fibers express high levels of TTX-R NaV1.8 and NaV1.9, and TTX-S NaV1.7 and NaV1.6. These differences in the Na+ channel expression are reflected in the differences in the morphology of the action potential waveform: NaV1.7 contributes to the rising phase of the action potential and amplifies subthreshold stimulation, while NaV1.8 contributes mainly to the rising phase and NaV1.9 amplifies subthreshold stimuli. Furthermore, NaV1.7, NaV1.8, and NaV1.9 have been reported to cause abnormal pain or painlessness in humans owing to gain-of-function or loss-of-function mutations [4].” (Pages 6–7:lines 57–70)

Ok

3) Description of the results should be better written.

Replace “Fig. X shows” throughout the section. For instance, on page 20 you should state “we investigated/examined c-Fos expression” instead of “we observed c-Fos expression” and then “we observed” or “this revealed” instead of “Fig.3 shows”.

**Response:** Thank you for your valuable comment. We have revised the text in the manuscript accordingly

**Changes in the text:**

“To confirm peripheral sensitization by prolonged optogenetic stimulation, we examined the blue LED light irradiation hind paw withdrawal test …in the NaV1.x–ChR2 mice (Fig 2).” (Pages 19–20: lines 219–224) “we investigated c-Fos expression in the secondary sensory neurons in the dorsal horn of the spinal cord. We observed c-Fos expression in the ipsilateral and contralateral dorsal horn of the lumber spinal cord after long term optogenetic stimulation (Fig 3).” (Page 21: lines 237–240)4) “We examined the analgesic effect of 10 and 100 mg/kg DS-1971a, which is a selective NaV1.7 inhibitor [15], … in the NaV1.7-ChR2, NaV1.8-ChR2 and NaV1.9-ChR2 mice (Fig 4).” (Page 23: lines 256–261)

Ok

4) On page 21, there is no introduction about the aim of the experiment. Why studying DS-1971a effect? Why focusing on NaV1.7 and not investigating the others? Why looking at the responses of the NaV1.8 and NaV1.9 mice after DS-1971a treatment if NaV1.7 is “globally expressed”? This also goes back to better describing the expression and role of these three sodium channels in the introduction. Your description of the results (lines 241-243) is a title of a figure legend but definitively not a result description/explanation. This must be rephrased. Additional experiments should also be done to fully support your conclusions mentioned in the discussion. It is important to examine the long-lasting effect of the NaV1.7 inhibitor using the same treatment paradigm. Given your model elicits allodynia for 24 h, does NaV1.7 inhibition prevent hypersensitivity appearance when looking at 24 h post-optogenetic stimulation or does it only block the acute phase you investigated here and simply delays sensory alterations?

In this line, NaV expressions are altered in neuropathic pain, it would be of interest to assess whether this is the case in your model 3 h, 24 h, and 48 h after light simulation during the acute phase when DS-1971a is effective, the subacute phase (to link it with the (lack of) effect of DS-1971a at that time point), and when normal sensory response is restored, respectively.

Additionally, given DS-1971a is efficient in all mouse genotypes, you would gain crucial information i/ by examining the influence of DS-1971a in hyperalgia tests in addition to allodynia and ii/ by specifically discussing the role of A-beta sensory fibres, and C vs A-delta nociceptive fibers as the three NaV channels have different expression patterns.

In this line, it would be important to test the impact of low and high TTX doses to discriminate A fibre (low dose) from A+C fibre (high dose) implications in nociceptive and sensory tests.

Also, does blocking NaV1.8 using VX-548 or LTGO-33 for instance lead to the same result in all three genotypes in different nociceptive and sensory tests?

This is crucial for the description of your model, for the understanding of the role of each sodium channel, thereby for proposing specific therapeutic targets for specific pain types.

**Response**: Thank you for your valuable comment. The purpose of the experiments with DS-1971a has been added. The description of the results has also been revised. NaV1.7 is abundantly distributed in DRG and is strongly associated with pain generation, and selective NaV1.7 inhibitors have often been studied for their analgesic effects in animal models of neuropathic pain. Fortunately, we were able to use DS-1971a in this study because Daiichi Sankyo gifted us DS-1971a.

Although DS-1971a suppressed neuropathic pain in NaV1.7-ChR2 mice as expected, it was surprising that DS-1971a also suppressed neuropathic pain in NaV1.8-ChR2 and NaV1.9-ChR2 mice. As described in Discussion, since NaV1.7 is globally expressed in various types of DRG, we hypothesize that NaV1.7 is also expressed in DRG expressing NaV1.8 and NaV1.9 and that DS-1971a also suppresses these DRG.

As the analgesic effect of DS-1971a on SNL and PSL mice in previous studies was around 2-4 h [15], in the present study, we believe that DS-1971a also temporarily alleviates neuropathic pain in our model. So, we did not observe any long-term effects of DS-1971a.

They authors did not observe or they did not examine? I still think it is an important experiment to see whether blocking the initial phase of nociception is sufficient to prevent central sensitization.

However, as reviewer 1 has indicated, it is possible that NaV expression is increased in our model of neuropathic pain, and initial administration of DS-1971a may provide long-term pain relief by suppressing it. To elucidate these issues, analysis of changes in NaV expression should also be performed. Therefore, we have not included these matters in this study and would like to leave them for future research.

Again DS-1971a long-term effect should be studied and depending on the results examining NaV expression changes may or may not be of importance.

Selective Na+ channel inhibitors against NaV1.7 and NaV1.8 exist, but there are currently no selective inhibitors against NaV1.9. As reviewer 1 has accurately pointed out, additional experiments with selective inhibitors against NaV1.8, such as VX-548, or with TTX would provide more insight, but due to cost and time constraints, we would like to limit the current report to experiments with DS-1971a. However, the fact that we were able to demonstrate the analgesic effect of DS-1971a on neuropathic pain is of great significance for future clinical practice.

I would have agreed if the authors had focused on NaV1.7 only but they examined the importance of several channels that could have different impact on the establishment of central sensitization. In the end they do not examine deep enough the importance of NaV1.7 nor do they investigate the importance of at least NaV1.8 (as NaV1.9 has no selective inhibitor).

As they point out DS-1971a could be of importance for clinical practice, but they cite a reference supporting this already, thus mellowing the importance of their results, and we circle back to a better understanding of their model in respect to clinical picture.

Experiments 1 and 3 are mandatory and experiment 2 remains optional depending on the result of experiment 1.

**Changes in the text:**

“Clinical trials of selective Na+ channel inhibitors are underway for the treatment of acute postoperative pain and neuropathic pain [17]. We have examined the analgesic effect of 10 and 100 mg/kg DS-1971a, which is a selective NaV1.7 inhibitor [15], on long-term optogenetic stimulation-induced neuropathic pain in NaV1.x–ChR2 mice. DS-1971a at both 10 mg/kg and 100 mg/kg almost completely suppressed long-term optogenetic stimulation-induced neuropathic pain in the NaV1.7-ChR2, NaV1.8-ChR2 and NaV1.9-ChR2 mice (Fig 4).” (Page 23: lines 255–261)

Minor comments

1) c-Fos expression is not a pain perception marker as stated several times in the manuscript. It reflects neuronal activity. In this case linked to nociceptor stimulation yet its expression is not specific to pain. This has therefore to be modified.

**Response:** Thank you for your valuable comment. We have modified the text as per your suggestion.

**Changes in the text:**

“c-Fos, a marker for neuronal activity following noxious stimulation, ” (Page 3: line 36) (Page 26: line 298)

OK

2) Below-mentioned sentences in introduction (lines 65-67) belong to the discussion or need to be rephrased:

“Previously, Daou et al. generated NaV1.8−ChR2 mice using the NaV1.8−Cre recombinase knock-in mouse line and reported results similar to ours [10]. They also reported the occurrence of transient neuropathic pain following long-term exposure to blue light.”

**Response:** Thank you for your valuable comment. We have rephrased the text as per your suggestion.

**Changes in the text:**

“Previously, Daou et al. reported the occurrence of transient neuropathic pain following long-term exposure to blue light in NaV1.8−ChR2 mice [11].” (Pages 7–8: lines 78–80)

OK

3) Materials & Methods, line 83. The authors mention that the experimenters are blinded to the mouse genotypes. Are they also blinded to the treatments (DS-1971a vs vehicle) used as this is also crucial?

**Response:** Thank you for your valuable comment. We have modified the text as per your suggestion.

**Changes in the text:**

“Mice in each group were randomly selected, and the experimenter was blinded to the mouse genotype and drug treatment.

” (Page 9: lines 95–97)

OK

4) Materials & Methods, lines 149-151. The below-mentioned sentence goes into the results section: “Mice were confirmed hypersensitive by von Frey test 1 hour after a prolonged (30 min), suprathreshold (7.5 mW for NaV1.7–ChR2, 1.8 mW for NaV1.8–ChR2, and 2.25 mW for NaV1.9–ChR2) light stimulation.”

**Response:** Thank you for your valuable comment. However, we believe that this text should be in the Materials & Methods section. Instead, the irradiation times have been added to figure legend.

**Changes in the text:**

“c-Fos expression in the ipsilateral and contralateral dorsal horn of the lumber spinal cord after long term (30 min) optogenetic stimulation (7.5 mW for NaV1.7–ChR2, 1.8 mW for NaV1.8–ChR2, and 2.25 mW for NaV1.9–ChR2) are shown in (a) NaV1.7–ChR2, (b) NaV1.8–ChR2, (c) NaV1.9–ChR2 mice.” (Page 21: line 245)

This is still not a description of the immunofluorescence protocol, but as they included the details in the figure legend, they can simplify by stating that mice that showed hypersensivity using the von Frey test were then fixed for immunofluorescence analysis.

5) Results. Line 208. “Continuous” should be replaced by “prolonged”.

**Response:** Thank you for your valuable comment. We have modified the text as per your suggestion.

**Changes in the text:**

“To confirm peripheral sensitization by prolonged optogenetic stimulation, we examined the blue LED light irradiation hind paw withdrawal test before and 1 hour after prolonged (30 min) optogenetic stimulation.” (Pages 19-20: lines 219-221)

OK

6) Discussion is lacking an introductory a summary of your findings.

**Response:** Thank you for your valuable comment. We have added the text as per your suggestion.

**Changes in the text:**

“In this study, we demonstrated that a reversible neuropathic pain state was established persisting for a minimum of 24 hours when light-responsive pain mice (NaV1.x-ChR2 mice) was irradiated with light of an intensity that consistently7) 8) elicited pain. Furthermore, the mice also showed hypersensitivity to light irradiation and mechanical stimulation. The c-Fos expression increased in the dorsal horn of the spinal cord on the light irradiated side. DS-1971a, a selective NaV1.7 inhibitor, was effective in attenuating neuropathic pain in all the light-responsive pain mice.”(Page 24: lines 273–279)

OK

7)Discussion (lines 276-279). This belongs to the result section.

**Response:** Thank you for your valuable comment. We have added and revised the texts as per your suggestion.

**Changes in the text:**

“DS-1971a at both 10 mg/kg and 100 mg/kg almost completely suppressed long-term optogenetic stimulation-induced neuropathic pain in the NaV1.7-ChR2, NaV1.8-ChR2 and NaV1.9-ChR2 mice (Fig 4).” (Page 23: lines 258–261)

“In this study, the selective NaV1.7 inhibitor DS-1971a for long-term optogenetic stimulation-induced neuropathic pain completely suppressed pain in the NaV1.x-ChR2 mice.” (Pages 26–27: lines 301–303)

OK

8) Discussion (line 286). Please give references.

**Response:** Thank you for your valuable comment. We have added a reference.

**Changes in the text:**

“…from these animal models [19].” (Page 27: line 313)

OK
