# Supplementary material for: Reversible neuropathic pain model created by long-term optogenetic nociceptor stimulation using light-responsive pain mice
Source: PLoS One. 2025 May 30;20(5):e0323628. doi: 10.1371/journal.pone.0323628 (PMC12124532; doi:10.1371/journal.pone.0323628)
Supplement: Attachment — Submitted filename: Response_to_Reviewers_auresp_2.docx [file pone.0323628.s005.docx]

Dear Dr. Julian Cheron,

Thank you for giving me the opportunity to submit a revised draft of the manuscript titled “**Reversible neuropathic pain model created by long-term optogenetic nociceptor stimulation using light-responsive pain mice.”** to *PLoS ONE* (Manuscript ID: PONE-D-24-58742).

We greatly appreciate the time and effort that you and the reviewers have dedicated to providing your valuable feedback on our manuscript. We are grateful to the reviewers for their insightful comments on this paper.

We have extensively revised our paper to reflect most of the suggestions provided by the reviewers. We have highlighted the changes within the manuscript in red font in the marked version (Revised manuscript with Track Changes). Here is a point-by-point response to the reviewers’ comments and concerns.

Reviewer: 1

Major comments:

1) The use of “neuropathic pain” in the description of their model is confusing. Indeed, neuropathy is large family of pain types which can be acute, a chronic state resulting from neuronal lesion with sensory alterations, combined to inflammation or metabolic alterations... In the discussion, they compare the advantage of their acute model of nociception over sciatic nerve injury that leads to chronic neuropathy, a very different clinical picture. A more precise description of the proposed model in respect to clinical relevance therefore needs to be clarified to draw the right conclusions.

In this line, the authors should give examples and references about the other animal models of neuropathic pain they are comparing theirs to (introduction, line 53) to clarify latter the potential added value and limitations of their model. Also, they refer to a previous work of theirs introducing this optogenetic-based model (introduction, line 61-65) and mention that “nociceptive pain was produced”, not neuropathic pain….

**Response:** Thank you for your valuable comment. We have already provided examples and references about the other animal models of neuropathic pain, which we are comparing our model to, to clarify latter the potential added value of our model in the discussion section. Kindly see Discussion, Pages 27–29: lines 311–327, “A multitude of animal models of neuropathic pain…specific voltage-gated Na^+^ channels”.

The authors did not include any example nor references of other models of neuropathic pain in the introduction (as previously asked) to later compare the supposed advantages of their model.

Indeed, in the discussion, they did not get into the specificities of their model vs others in respect to the fact that existing models “may not accurately recapitulate the clinical manifestations of the disease”. I still do not see how their model addresses this question. I understand their model produces central sensitization (which may lead to neuropathic pain) and that the model can be of importance for studying the initial mechanisms of central sensitization, but there is no data supporting that this is a clinically relevant model of neuropathic pain, notably because neuropathic pain is a chronic state (for instance in the Chung model which is the only one they discuss) while sensory alterations are reversible after 24 hours in their model of acute neuropathy.

Our light-responsive pain mice (Na_V_1.x−ChR2) induce nociceptive pain on exposure to specific wavelengths, similar to the light-responsive pain mice (Na_V_1.8−ChR2) developed by Daou et al. Moreover, repeated nociceptive pain can lead to neuropathic pain due to central sensitization [16]. Daou et al. and we have also demonstrated that prolonged light exposure can cause neuropathic pain. We have already mentioned these points in the discussion section (Page 26: lines 294-301, “Daou et al. reported that…thereby leading to central sensitization [16]”).

**Response 2:** Thank you for your valuable comment. Conventional animal models of neuropathic pain in which peripheral nerves are physically damaged may result in motor neuropathy and sensory neuropathy other than pain. Optogenetics-based neuropathic pain models are superior to conventional animal models in that they can precisely target pain nerves. Furthermore, by targeting only the pain nerves, they may more faithfully reproduce the clinical manifestations of neuropathic pain. The text has been added in light of the above.

**Changes in the text:**

“The most widely employed models of neuropathic pain attributed to peripheral neuropathy in rodents are physical injury models of peripheral nerves, including the spared nerve injury (SNI), chronic constriction injury (CCI), partial sciatic nerve ligation (PSL), partial sciatic nerve ligation (PSL), and spinal nerve ligation (SNL) (Chung model) models [3]. Although these models produce allodynia, they can cause motor and/or non-pain sensory neuropathies.” (Page 5: lines 52–57)

“Such optogenetics-based neuropathic pain models are superior to conventional animal models in that they can target only the sensory nerves responsible for pain.” (Page 8: lines 85–87)

“Furthermore, although pain persisted for up to 24–36 hours in this study, more chronic neuropathic pain may occur with higher light intensity or longer exposure duration, which may contribute to the understanding of chronic pain.” (Page 30: lines 342–344)

4) On page 21, there is no introduction about the aim of the experiment. Why studying DS-1971a effect? Why focusing on Na_V_1.7 and not investigating the others? Why looking at the responses of the Na_V_1.8 and Na_V_1.9 mice after DS-1971a treatment if Na_V_1.7 is “globally expressed”? This also goes back to better describing the expression and role of these three sodium channels in the introduction. Your description of the results (lines 241-243) is a title of a figure legend but definitively not a result description/explanation. This must be rephrased.

Additional experiments should also be done to fully support your conclusions mentioned in the discussion. It is important to examine the long-lasting effect of the Na_V_1.7 inhibitor using the same treatment paradigm. Given your model elicits allodynia for 24 h, does Na_V_1.7 inhibition prevent hypersensitivity appearance when looking at 24 h post-optogenetic stimulation or does it only block the acute phase you investigated here and simply delays sensory alterations?

In this line, Na_V_ expressions are altered in neuropathic pain, it would be of interest to assess whether this is the case in your model 3 h, 24 h, and 48 h after light simulation during the acute phase when DS-1971a is effective, the subacute phase (to link it with the (lack of) effect of DS-1971a at that time point), and when normal sensory response is restored, respectively.

Additionally, given DS-1971a is efficient in all mouse genotypes, you would gain crucial information i/ by examining the influence of DS-1971a in hyperalgia tests in addition to allodynia and ii/ by specifically discussing the role of A-beta sensory fibres, and C vs A-delta nociceptive fibers as the three Na_V_ channels have different expression patterns.

In this line, it would be important to test the impact of low and high TTX doses to discriminate A fibre (low dose) from A+C fibre (high dose) implications in nociceptive and sensory tests.

Also, does blocking Na_V_1.8 using VX-548 or LTGO-33 for instance lead to the same result in all three genotypes in different nociceptive and sensory tests?

This is crucial for the description of your model, for the understanding of the role of each sodium channel, thereby for proposing specific therapeutic targets for specific pain types.

**Response**: Thank you for your valuable comment. The purpose of the experiments with DS-1971a has been added. The description of the results has also been revised.

Na_V_1.7 is abundantly distributed in DRG and is strongly associated with pain generation, and selective Na_V_1.7 inhibitors have often been studied for their analgesic effects in animal models of neuropathic pain. Fortunately, we were able to use DS-1971a in this study because Daiichi Sankyo gifted us DS-1971a.

Although DS-1971a suppressed neuropathic pain in Na_V_1.7-ChR2 mice as expected, it was surprising that DS-1971a also suppressed neuropathic pain in Na_V_1.8-ChR2 and Na_V_1.9-ChR2 mice. As described in Discussion, since Na_V_1.7 is globally expressed in various types of DRG, we hypothesize that Na_V_1.7 is also expressed in DRG expressing Na_V_1.8 and Na_V_1.9 and that DS-1971a also suppresses these DRG.

As the analgesic effect of DS-1971a on SNL and PSL mice in previous studies was around 2-4 h [15], in the present study, we believe that DS-1971a also temporarily alleviates neuropathic pain in our model. So, we did not observe any long-term effects of DS-1971a.

They authors did not observe or they did not examine? I still think it is an important experiment to see whether blocking the initial phase of nociception is sufficient to prevent central sensitization.

However, as reviewer 1 has indicated, it is possible that Na_V_ expression is increased in our model of neuropathic pain, and initial administration of DS-1971a may provide long-term pain relief by suppressing it. To elucidate these issues, analysis of changes in Na_V_ expression should also be performed. Therefore, we have not included these matters in this study and would like to leave them for future research.

Again DS-1971a long-term effect should be studied and depending on the results examining NaV expression changes may or may not be of importance.

Selective Na^+^ channel inhibitors against Na_V_1.7 and Na_V_1.8 exist, but there are currently no selective inhibitors against Na_V_1.9. As reviewer 1 has accurately pointed out, additional experiments with selective inhibitors against Na_V_1.8, such as VX-548, or with TTX would provide more insight, but due to cost and time constraints, we would like to limit the current report to experiments with DS-1971a. However, the fact that we were able to demonstrate the analgesic effect of DS-1971a on neuropathic pain is of great significance for future clinical practice.

I would have agreed if the authors had focused on NaV1.7 only but they examined the importance of several channels that could have different impact on the establishment of central sensitization. In the end they do not examine deep enough the importance of NaV1.7 nor do they investigate the importance of at least NaV1.8 (as NaV1.9 has no selective inhibitor).

As they point out DS-1971a could be of importance for clinical practice, but they cite a reference supporting this already, thus mellowing the importance of their results, and we circle back to a better understanding of their model in respect to clinical picture.

Experiments 1 and 3 are mandatory and experiment 2 remains optional depending on the result of experiment 1.

**Changes in the text:**

“Clinical trials of selective Na^+^ channel inhibitors are underway for the treatment of acute postoperative pain and neuropathic pain [17]. We have examined the analgesic effect of 10 and 100 mg/kg DS-1971a, which is a selective Na_V_1.7 inhibitor [15], on long-term optogenetic stimulation-induced neuropathic pain in Na_V_1.x–ChR2 mice. DS-1971a at both 10 mg/kg and 100 mg/kg almost completely suppressed long-term optogenetic stimulation-induced neuropathic pain in the Na_V_1.7-ChR2, Na_V_1.8-ChR2 and Na_V_1.9-ChR2 mice (Fig 4).” (Page 23: lines 255–261)

**Response 2:** Thank you for your valuable comment. As neuropathic pain in light-responsive pain mice is reversible, over time it becomes impossible to determine whether the analgesia is due to DS-1971a or to a natural process. Furthermore, the effects of DS-1971a last for 2–4 hours. Therefore, I have only observed the effects of DS-1971a for a short time immediately after the onset of neuropathic pain in this study. And unfortunately, I do not currently have temporary possession of the Na_V_1.x-ChR2 mice due to a change of laboratory in April this year. It is also expected that it will take some time to set up the new laboratory. Therefore, while we fully appreciate that the additional experiments recommended by reviewer would make our study more meaningful, additional experiments could not be performed. Thus, I hope that additional experiments on the long-term effects of DS-1971a and selective Na_V_1.8 inhibitors could be left for future research. These have been added to the Discussion section as limitations to this study.

**Changes in the text:**

“In the present study, we only observed the short-term analgesic effects of DS-1971a; however, successful initial treatment of neuropathic pain may lead to long-term analgesic effects. Furthermore, to understand the role of Na_V_1.8 in neuropathic pain, it is important to clarify how selective Na_V_1.8 inhibitors (although no selective Na_V_1.9 inhibitors currently exist) act in the respective Na_V_1.x-ChR2 mice. These limitations of the present study and issues should be addressed in future studies.” (Page 28: lines 319–324)

Minor comments

4) Materials & Methods, lines 149-151. The below-mentioned sentence goes into the results section:

“Mice were confirmed hypersensitive by von Frey test 1 hour after a prolonged (30 min), suprathreshold (7.5 mW for Na_V_1.7–ChR2, 1.8 mW for Na_V_1.8–ChR2, and 2.25 mW for Na_V_1.9–ChR2) light stimulation.”

**Response:** Thank you for your valuable comment. However, we believe that this text should be in the Materials & Methods section. Instead, the irradiation times have been added to figure legend.

**Changes in the text:**

“c-Fos expression in the ipsilateral and contralateral dorsal horn of the lumber spinal cord after long term (30 min) optogenetic stimulation (7.5 mW for Na_V_1.7–ChR2, 1.8 mW for Na_V_1.8–ChR2, and 2.25 mW for Na_V_1.9–ChR2) are shown in (a) Na_V_1.7–ChR2, (b) Na_V_1.8–ChR2, (c) Na_V_1.9–ChR2 mice.” (Page 21: line 245)

This is still not a description of the immunofluorescence protocol, but as they included the details in the figure legend, they can simplify by stating that mice that showed hypersensivity using the von Frey test were then fixed for immunofluorescence analysis.

**Response 2:** Thank you for your valuable comment. We have added the text in the Result section as per your suggestion.

**Changes in the text:**

“Mice that demonstrated hypersensitivity using the von Frey test were fixed for immunofluorescence analysis.” (Page 22: lines 245–247)
